# Higher serum apolipoprotein B level will reduce the bone mineral density and increase the risk of osteopenia or osteoporosis in adults

**DOI:** 10.3389/fcell.2022.1054365

**Published:** 2022-12-09

**Authors:** RunJiu Zhu, Yuan Xu, ZhaoFu Wang, Hui Li, MingRui Song, HaoYang Wan, Hong Yang, Xin Zhang, Yu Chai, Bin Yu

**Affiliations:** ^1^ Division of Orthopaedics and Traumatology, Department of Orthopaedics, Nanfang Hospital, Southern Medical University, Guangzhou, China; ^2^ Guangdong Provincial Key Laboratory of Bone and Cartilage Regenerative Medicine, Nanfang Hospital, Southern Medical University, Guangzhou, China; ^3^ Department of Orthopaedics, People’s Hospital of Ningxia Hui Autonomous Region, Yinchuan, China; ^4^ Department of Orthopaedics & Traumatology, Wuyi Hospital of Traditional Chinese Medicine, Jiangmen, China; ^5^ Department of Respiratory and Critical Care Medicine, Nanfang Hospital, Southern Medical University, Guangzhou, China

**Keywords:** osteoporosis, apolipoprotein B, bone mineral density, cross-sectional study, NHANES

## Abstract

**Objective:** There is very limited evidence in the NHANES database linking serum apolipoprotein B and lumbar bone mineral density (BMD) in adults aged 20–59 years. There are few studies associating apolipoprotein B concentrations with BMD, and there is some debate about the association between obesity and BMD. Therefore, the purpose of this study was to determine the association between serum apolipoprotein B concentrations and lumbar spine BMD in adults aged 20–59 years and to predict its association with risk of osteopenia or osteoporosis.

**Methods:** A cross-sectional study of the entire US ambulatory population was conducted using data from the National Health and Nutrition Examination Survey (NHANES) database. Weighted multiple regression equation models were used to assess the association between serum apolipoprotein B and lumbar BMD. A logistic weighted regression model was used to assess the association between serum apolipoprotein B concentrations and risk of osteopenia or osteoporosis. Subsequent stratified analyses were performed to refine the primary population of association.

**Results:** Our study showed a significant negative association between serum apolipoprotein B concentration and lumbar BMD and a significant positive association with the risk of osteoporosis or osteopenia in the total population. After stratifying by sex, age and race, we concluded differently. The association of serum apolipoprotein B concentration with lumbar spine BMD and risk of osteopenia or osteoporosis was significant in male, but not in female. After stratification by age, the negative association between serum apolipoprotein B concentrations and lumbar BMD and the positive association with risk of osteopenia or osteoporosis was more significant in the 30–39 and 50–59 years age groups. When stratified by race, serum apolipoprotein B concentrations were significantly negatively associated with lumbar BMD and positively associated with risk of osteopenia or osteoporosis in Mexican American and non-Hispanic black populations. Thus, these findings suggest that these associations are influenced by sex, age, and race, respectively.

**Conclusion:** Our results suggest that the association between serum apolipoprotein B levels and the risk of lumbar BMD and osteopenia or osteoporosis varies by sex, age, and race. In men, elevated serum apolipoprotein B levels were negative for bone quality. Elevated serum apolipoprotein B levels in the age groups 30–39 and 50–59 years also had a negative effect on bone quality. In the Mexican American and Non-Hispanic Black populations, elevated serum apolipoprotein B levels also had a significant negative effect on bone quality.

## 1 Introduction

Osteoporosis (OP) is a systemic disease affecting a large proportion of the world’s population (Sanchez-Riera et al., 2014; Cauley, 2018). The main features of osteoporosis include reduction in the bone mineral density, impairment of bone strength, and destruction of its fine structure, resulting in an increased risk of fracture at the involved site (Castaneda et al., 2020; Wilson-Barnes et al., 2022). Therefore, a reduction in bone mineral density can be used as a common indicator for the diagnosis of osteoporosis (Kemp et al., 2017; Yang et al., 2020). In addition, BMD obtained by dual-energy X-ray absorptiometry (DXA) can be used to diagnose osteoporosis, assess the risk of developing fragility fractures (Medical Advisory, 2006; Chen et al., 2022), track changes in the severity of osteoporosis, and assess the effects of certain habit changes or efficacy of medications (Lopez-Peralta et al., 2022). Osteoporosis is mainly divided into primary and secondary osteoporosis (Miyakoshi et al., 2017).

Women are generally at greater risk of developing osteoporosis than men (Rezaei and Dragomir-Daescu, 2015). Most men tend to have larger and stronger bones than women and usually lose less bone mass over their lifetime (Lambert et al., 2011; Rezaei and Dragomir-Daescu, 2015). The incidence of fractures due to osteoporosis is usually lower in men than in women (Duan et al., 2001). However, secondary osteoporosis, such as alcoholic osteoporosis, is more common in men (Patsch et al., 2011; Pasco et al., 2021). The dangers of osteoporosis are not just the symptoms, but also the complications it creates (Li et al., 2022; Shen et al., 2022; Wang and Xiao, 2022). Researchers generally evaluate the development of osteoporosis by measuring bone mineral density at the lumbar spine (Genest et al., 2021), but this is a physical test to detect bone health. Therefore, it is essential to investigate new biomarkers of bone health, such as serum apolipoprotein B. In previous studies, obesity lead to an increase in bone mineral density because of the higher mechanical load, which may contribute in protecting the bone (Gkastaris et al., 2020; Qiao et al., 2020). It has also been observed that adult obese patients have higher bone mineral density in the lumbar spine and femoral neck than healthy weight individuals. In general, obesity is negatively associated with femoral neck osteoporosis suggesting that obesity is a protective factor for osteoporosis in some cases (Qiao et al., 2020). However, additional studies have found a significant negative association between fat mass and BMD, particularly in men (Kim et al., 2010). These findings contradict studies suggesting that obesity causes increased BMD.

Serum apolipoprotein B concentration is a key indicator for the diagnosis of hyperlipidemia. Thus, serum apolipoprotein B concentrations could be an important indicator of changes in BMD(Ahmed et al., 2018; Qu et al., 2021; Nelson et al., 2022). In cardiovascular disease, for example, apolipoprotein B levels have become a key player in atherosclerosis. However, the relationship between serum apolipoprotein B level and bone health has not been well studied. Some studies have found that elevated dyslipidemia leads to lower bone turnover markers in patients with type 2 diabetes, but that there is a positive correlation between the LDL cholesterol/apolipoprotein B ratio and bone turnover markers in patients with type 2 diabetes (Lu et al., 2022). However, the relationship between serum apolipoprotein B concentrations and bone turnover has not been investigated in depth. Only a few studies have investigated the mechanism underlying this association with BMD. Therefore, the present study on the association between apolipoprotein B levels and BMD is essential. We demonstrated not only the correlation between serum apolipoprotein B concentration and bone mineral density, but also the correlation between changes in serum apolipoprotein B concentration on the risk of osteopenia or osteoporosis. This suggests a new biomarker for monitoring changes in BMD and predicting the development of osteopenia or osteoporosis. The National Health and Nutrition Examination Survey database (NHANES) is a good source of data collection, and provides basis for the study.

## 2 Materials and methods

### 2.1 Data and study population

In the current study, the data used for analysis came from the National Health and Nutrition Examination Survey database (NHANES). We collected and statistically analyzed the survey data between 2011 and 2016. This is a probability sample of the non-institutionalized US population. The data has been collected every 2 years since 1999 from a nationally representative sample. These surveys were supervised and administered by the National Center for Health Statistics (NCHS), and included basic demographics, laboratory tests, questionnaires, and basic physical measurements. The sample data were stratified into multi-stage and complex probability samples. These cross-sectional data can be used to assess the health and nutritional status of the US non-institutionalized population, and by collating and analyzing the data from this database, we can explore the factors associated with disease so that the onset and progression of disease can be prevented. The advantage of this database is that researchers worldwide can download data and analyze the factors freely. The total population of this study was 29,902.

**FIGURE 1:**
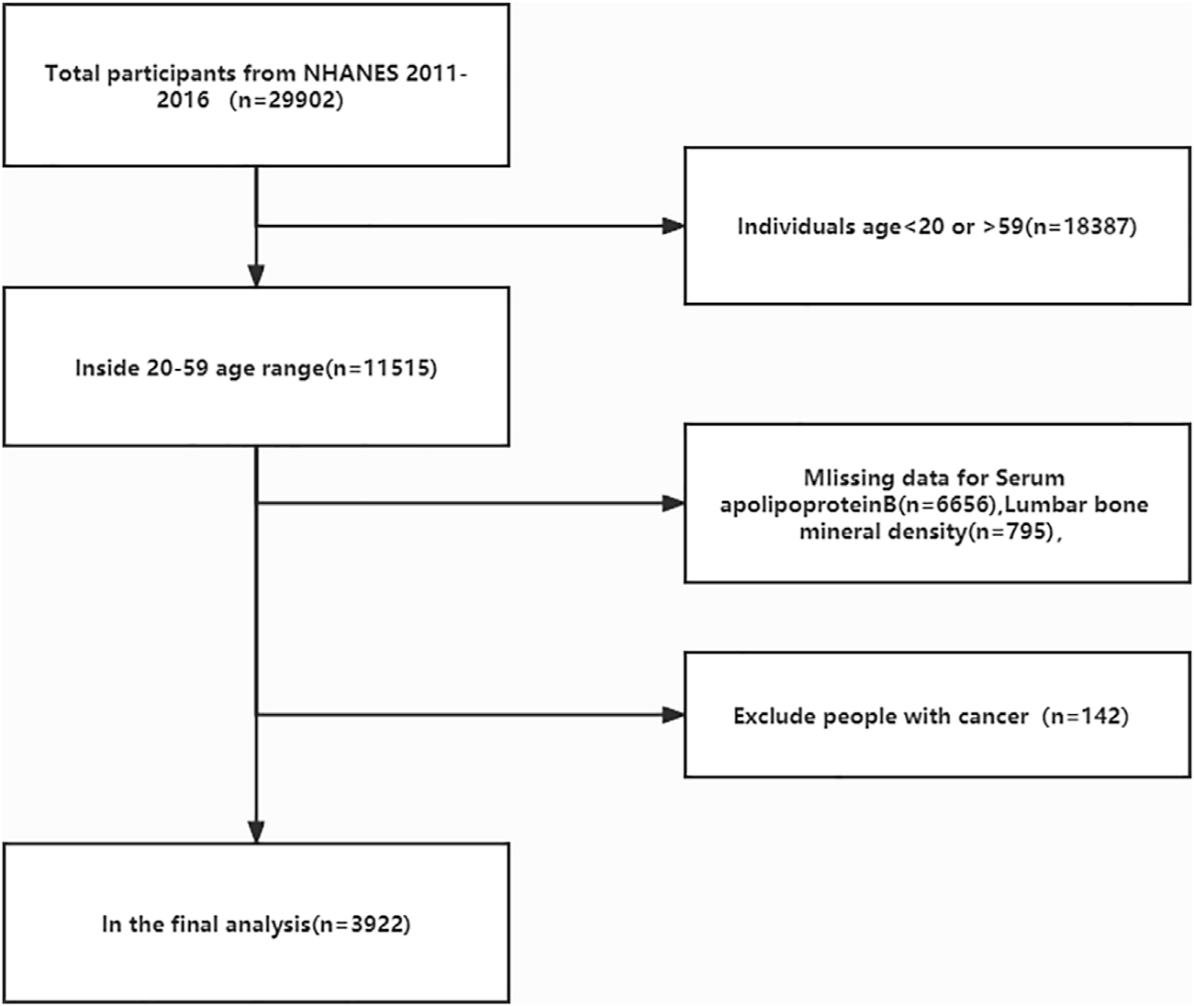
Flow diagram of the inclusion criteria and exclusion criteria. NHANES, National Health and Nutrition Examination Survey.

The age of the study population was chosen because adolescents are in the bone constructing stage before the age of 20 years owing to their growth spurt, and a review of previous literature on BMD studies found that most were between the ages of 20–59 years (Bourne et al., 2017; Zhu et al., 2021a; Reesor-Oyer et al., 2021). We excluded this group because not only were there more underlying diseases in the >60 years age group, but the database did not fully cover all diseases in this group, making it difficult to exclude confounding factors. We also excluded the cancer group from the study population because cancer can also lead to changes in apolipoprotein B concentrations and bone mass (Avall-Lundqvist and Peterson, 1996; Castaneda et al., 2022). The current study was limited to survey participants aged 20–59 years (n = 11,515). After excluding apolipoprotein B deficient data (n = 6,656), lumbar spine BMD deficient data (n = 795), and cancer patients (n = 142), 3,922 subjects were included in the final analysis (Figure 1). The NCHS ethical review committee approved the conduct of the NHANES database cross-sectional study, and all participants signed a written informed consent form.

### 2.2 Diagnosis of osteoporosis and osteopenia

All participants in the study had their bone mineral density (BMD) measured using dual-energy X-ray densitometry (DXA), and all measurements were analyzed using APEX software for lumbar spine scans, with additional details provided on the NHANES website. Same sex 20 to 29-year-old subjects were selected as the reference group for T-score calculation (Lv et al., 2022). A T-score ≥ -1 represented normal bone mass, a T-score of -1 to -2.5 represented reduced bone mass, and a T-score ≤ -2.5 was considered osteoporotic (Looker et al., 1998; Ensrud and Crandall, 2017). All participants were divided into two groups: the normal and osteoporosis or osteopenia.

### 2.3 Apolipoprotein B selection and analysis

Apolipoprotein B in the human serum can undergo an immunochemical reaction to form immune complexes. The complex changes the intensity of the scattered light by scattering the beam that passes through it, which allows us to measure the intensity of the scattered light on a Siemens Prospec chemistry analyzer to derive the apolipoprotein B concentration. Apolipoprotein B data are available from the NHANES dataset (http://cdc.gov/nchs/nhanes). In this study, apolipoprotein B concentrations were treated as continuous and categorical variables, multiple linear regression equations were used to determine the relationship between apolipoprotein B and BMD, and logistic regression equations were used to calculate the risk association between changes in serum apolipoprotein B concentrations and osteopenia or osteoporosis.

### 2.4 Covariates

Because of the potential influence of other factors on bone mineral density at this age, we selected appropriate covariates in the NHANES database by reviewing previous literature (Zhu et al., 2021a; Ye et al., 2021; Yang et al., 2022). Among the demographic data, the covariates were age (20–59 years), sex, race, and income level (Zhu et al., 2021a). Age was subdivided into–20–29, 30–39, 40–49 and 50–59 age groups, and race was subdivided into Mexican American, other Hispanic, non-Hispanic white, non-Hispanic black, and other races. We selected BMI as a covariate in the inspection data list (Zhu et al., 2020). The covariates were serum urea nitrogen, total protein, serum uric acid, serum sodium, serum potassium, serum phosphorus, and serum calcium concentrations (Pan et al., 2020; Yao et al., 2020). In the questionnaire data, smoking status and daily activity were considered as potential covariates in this study (Zhu et al., 2021a; Zhu et al., 2021b).

### 2.5 Data analysis

All data analyses in this study were performed using R software (version 4.0.3; https://www.R-project.org), EmpowerStats (version 2.0; http://www.empowerstats.com), and Stata (version 16 http://www.stata.com). Data were considered statistically significant at *p* < 0.05. This study was based on participants with complete data in the database. Those with missing data for the independent variable apolipoprotein B and the dependent variable lumbar spine BMD were excluded from the analysis. All estimates in the data analysis were calculated by weighting the sample weights in the NHANES database, with those used in the study being one-third of the full sample weights according to the NHANES database weight selection criteria.

The study began with a statistical description of the entire population. Continuous variables were described using weighted means and standard deviations (SD) for baseline characteristics (Wang et al., 2022). Categorical variables were described using a weighted chi-square test, and regression coefficients (*β*) and 95% confidence intervals (CI) were calculated from weighted multiple linear regression equations to analyze the association between apolipoprotein B and BMD(Tang et al., 2022). The odds ratio (OR) and 95% confidence intervals (CI) were calculated using weighted univariate and multivariate logistic regression models (Wang et al., 2022), and the results were used to assess the relationship between serum apolipoprotein B concentrations and the risk of osteopenia or osteoporosis. Model 1 of the two regression models did not adjust for covariates; model 2 adjusted for three covariates: sex, age, and race; and model 3 adjusted for all covariates (Yang et al., 2022).

In the study, the aim was to analyze the association between serum apolipoprotein B and BMD in different groups by sex, age, and race, as well as analyze the relationship between the risk of osteopenia and osteoporosis. Thus, the population was analyzed by subgroups of sex, age group, and ethnicity, and the association between serum apolipoprotein B and BMD was explored using stratified weighted multiple linear regression equations, while the association between apolipoprotein B and the risk of osteopenia or osteoporosis was explored using stratified logistic regression equations. The nonlinear relationship between apolipoprotein B and BMD in the subgroup analysis was described by smoothed curve fitting.

## 3 Results

### 3.1 Participants’ baseline characteristics

In the current study, 29,902 participants were initially extracted from the NHANES database (2011–2012: n = 9,756, 2013–2014: n = 10,175, 2015–2016, n = 9,971). First, we excluded people who did not meet the age cutoff, that is, those younger than 20 years and older than 59 years (n = 18,387); those with missing serum apolipoprotein B data (n = 6,656), and those with missing lumbar spine BMD data (n = 795). We then excluded those diagnosed with cancer (n = 142) because of the large effect of tumors on serum apolipoprotein B and bone density. Data from 3,922 participants were ultimately included in the final analysis (Figure 1). Table 1 compares the baseline characteristics of the normal population and those with osteopenia or osteoporosis. Compared to the normal population, those with osteopenia or osteoporosis were older (*p* < 0.00001), less active (*p* < 0.00001), were smokers (*p* = 0.00734), and had lower income (2.684 ± 1.635 vs. 2.862 ± 1.624, *p* = 0.00907) than the normal population. Women had higher rates of osteopenia and osteoporosis than men (*p* = 0.03111). The proportion of people with osteopenia or osteoporosis was higher in Mexican Americans, other Hispanics, and other races at 13.653%, 9.097%, and 10.153%, respectively. Those with osteopenia or osteoporosis had higher serum apolipoprotein B concentrations (0.946 ± 0.256 vs. 0.902 ± 0.250, *p* = 0.00002) and lower BMD (0.835 ± 0.058 vs. 1.078 ± 0.121, *p* < 0.00001) than that in the normal population.

**TABLE 1:** Weighted characteristics of the study population.

	Normal (n=3137)	OP or Osteopenia (n=785)	*p*-value
Age (years)	38.500± 11.530	41.474 ± 11.754	0.00001
Sex (%)			0.03111
Men	53.102	48.684	
Women	46.898	51.316	
Race/ethnicity (%)			<0.00001
Mexican American	9.538	13.653	
Other Hispanic	6.859	9.097	
Non-Hispanic White	61.664	60.139	
Non-Hispanic Black	13.329	6.957	
Other Race	8.610	10.153	
Vigorous recreational activities (%)			<0.00001
Yes	33.317	23.850	
No	66.683	76.150	
Smoked at least 100 cigarettes in life (%)			0.00734
Yes	40.580	44.693	
'No	59.420	55.133	
Income to poverty ratio	2.862 ± 1.624	2.684 ± 1.635	0.00907
Blood urea nitrogen (mmol/L)	4.442 ± 1.523	4.460 ± 1.582	0.77447
Serum calcium (mmol/L)	2.335 ± 0.081	2.332 ± 0.083	0.40475
Serum potassium (mmol/L)	3.990 ± 0313	3.978 ± 0.312	032984
Serum sodium (mmo1/L)	139.021 ± 1.982	139.044 ± 2.169	0.78488
Serum phosphorus (mmol/L)	1.186± 0.174	1.209 ± 0.177	0.00156
Total protein (g/L)	71.301 ± 4.255	71.046 ± 4.357	0.15710
Serum uric acid(µmol/L)	323.490 ± 82.011	315.759 ± 75.017	0.02206
Body mass index (kg/m^2^)	29.125 ± 7.065	28.926 ± 6.396	0.49307
Serum apolipoproteinB (g/L)	0.902 ± 0.250	0.946 ± 0.256	0.00002
Lunbar bone mineral density (g/cm^2^)	1.078 ± 0.121	0.835 ± 0.058	<0.00001

The *p*-value for comparison of characteristics between Normal and OP or Osteopenia; Mean +/- SD for: Age, Income to poverty ratio, Blood urea nitrogen, Serum calcium, Serum potassium, Serum sodium , Serum phosphorus, Total protein, Serum uric acid, Body mass index, Serum apolipoproteinB, Lumbar bone mineral density % for: Sex Race/ethnicity, Vigorous recreational activities, Smoked at least 100 cigarettes in life.

### 3.2 Relationship between serum apolipoprotein B and lumbar spine bone mineral density

#### 3.2.1 Overall

**FIGURE 2:**
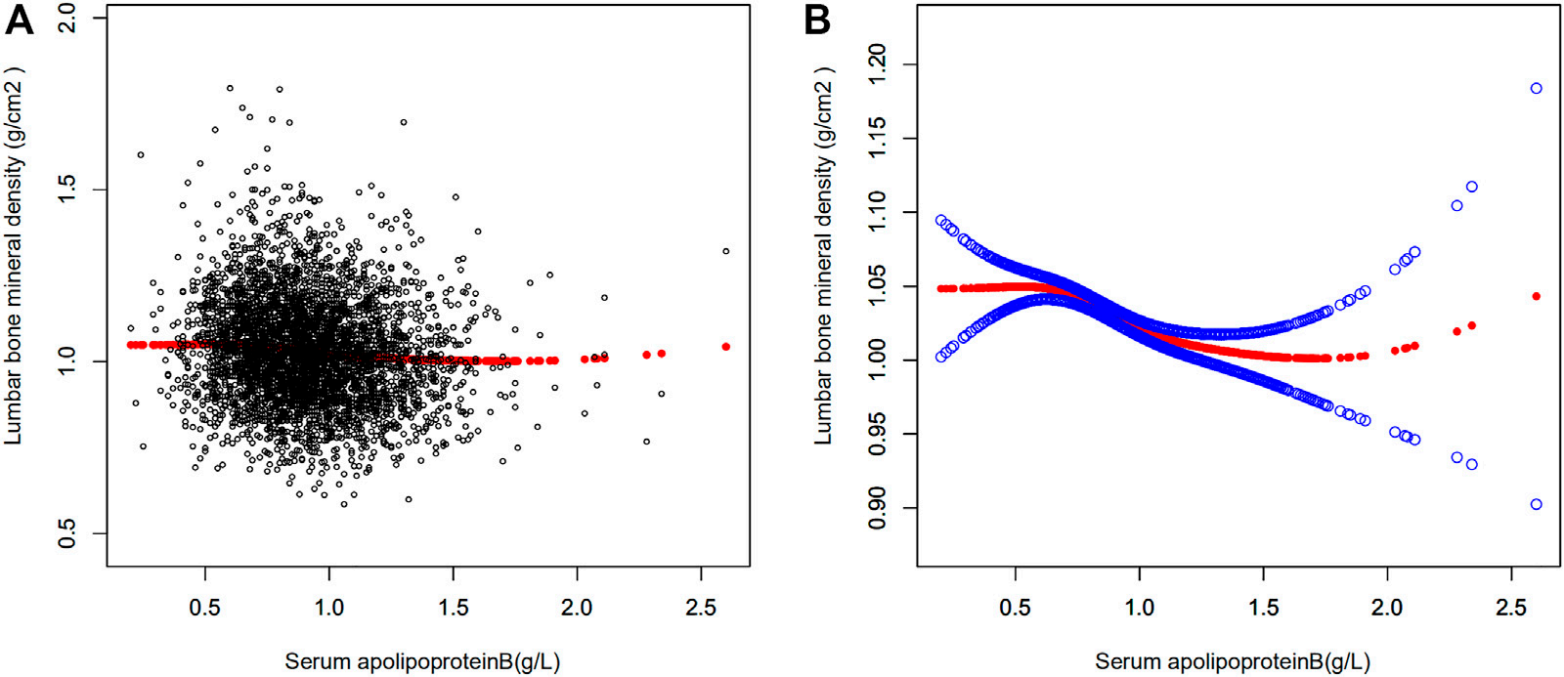
Relationship between serum apolipoprotein B and bone mineral density. **(A)** Each black dot represents a sample. **(B)** Red curves represent smoothed curves between variables, the blue bands represents the 95% confidence interval of the fitted curve (adjusted for age, race/ethnicity, sex, vigorous recreational activities, smoked at least 100 cigarettes in life, income to poverty ratio, blood urea nitrogen, serum calcium, serum potassium, serum sodium, serum phosphorus, total protein, serum uric acid, body mass index).

Multiple regression equations were used to analyze the correlation between serum apolipoprotein B concentration and BMD in the population. Table 2 shows the correlation between serum apolipoprotein B level and BMD when used as a continuous variable. The results presented in the three models of the multiple regression equation analysis showed a negative association between serum apolipoprotein B concentration and lumbar spine BMD. After adjusting for all covariates (Model 3), serum apolipoprotein B concentration was negatively and significantly associated with lumbar BMD (*β* = -0.05; 95% CI: −0.070, -0.030; *p* < 0.00001). Smoothed curves of serum apolipoprotein B concentration and lumbar spine BMD are shown in Figure 2.

**TABLE 2:** Associations of the Serum apolipoproteinB with lumbar bone mineral density.

	Model 1	Model 2	Model 3
	β (95% CI) *p* value	β (95% CI) *p* value	β (95% CI) *p* value
Serum apolipoproteinB (g/L)	−0.061(−0.080, −0.043) < 0.00001	−0.045 (−0.064, −0.026) < 0.00001	−0.050(−0.070,−0.030) < 0.00001
Serum apolipoproteinB (g/L ) categories				
Q1 (≤ 0.73)	Reference	Reference	Reference
Q2 (> 0.73, ≤ 0.89)	−0.021 (−0.035, −0.008) 0.00147	−0.016(−0.029, −0.003) 0.01586	−0.017(−0.030, −0.004) 0.00929
Q3 (> 0.89, ≤ 1.07)	−0.031 (−0.044, −0.018) < 0.00001	−0.022(−0.035, −0.009) 0.00125	−0.025(−0.038, −0.012) 0.00024
Q4 (> 1.07)	−0.045 (−0.058, −0.032) < 0.00001	−0.033(−0.047, −0.019) < 0.00001	−0.036 (−0.050, −0.022) < 0.00001
P trend	< 0.001	< 0.001	< 0.001

Model 1: no covariates were adjusted.

Model 2: Age, Race/ethnicity and Sex were adjusted.

Model 3: Age, Race/ethnicitv, Sex, Vigorous recreational activities, Smoked at least 100 cigarettes in lie Income to poverty ratio, Blood urea nitrogen, Serum calcium, Serum potassium, Serum sodium, Serum phosphorus, Total protein Serum uric acid, Body mass index.

Serum apolipoprotein B concentrations were transformed from continuous variables to categorical variables (Q1: ≤0.73 g/L,Q2: >0.73, ≤0.89 g/L,Q3: >0.89, ≤1.07 g/L,Q4: >1.07 g/L) to investigate the correlation between the two in different concentration intervals. A trend test (P trend) was also performed on the categorical variables of serum apolipoprotein B concentration and BMD to investigate the trend of decreasing BMD in the population with increasing serum apolipoprotein B concentration. As the P trend test in Table 2 was <0.001, there was a significant trend towards a decrease in lumbar spine BMD with increasing serum apolipoprotein B concentrations. The results of the analysis of the three models for Q2,Q3, and Q4 concentration intervals were obtained by regression analysis using the Q1 group as a control. The results suggested a negative correlation between serum apolipoprotein B concentration and lumbar spine BMD at each concentration interval after adjusting for all covariates (Q2: β = -0.017, 95% CI: −0.030, -0.004; *p* = 0.00929; Q3: β = -0.025, 95% CI: −0.038, -0.012, *p* = 0.00024; Q4: β = -0.036, 95% CI: −0.050, -0.022, *p* < 0.00001). This showed a significant negative correlation between serum apolipoprotein B and lumbar spine BMD in the Q2, Q3, and Q4 concentration intervals (Q2, *p* = 0.00929; Q3, *p* = 0.00024; Q4, *p* < 0.00001).

#### 3.2.2 Stratified analysis by sex, age, and race

**FIGURE 3:**
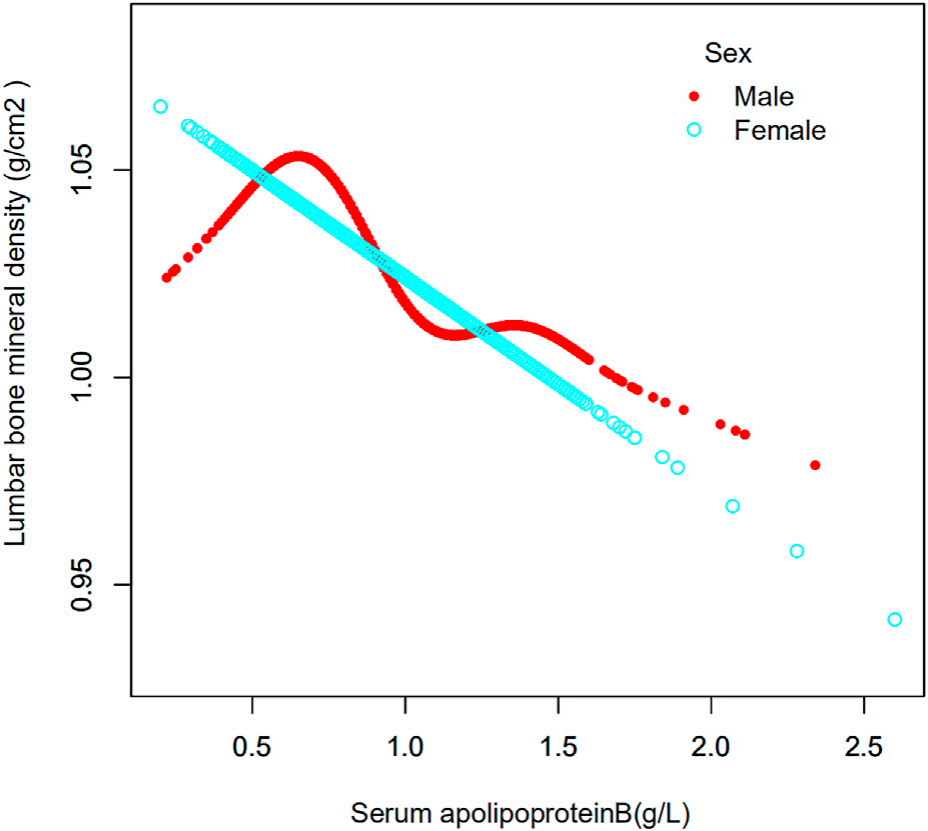
Association between serum apolipoprotein B concentrations and lumbar bone mineral density stratified by sex. Age, race/ethnicity, sex, vigorous recreational activities, smoked at least 100 cigarettes in life, income to poverty ratio, blood urea nitrogen, serum calcium, serum potassium, serum sodium, serum phosphorus, total protein, serum uric acid, body mass index were adjusted.

In this study, the population was stratified according to sex (Table 3). Serum apolipoprotein B concentrations were also divided into continuous and categorical variables for correlation analysis. In the fully adjusted covariate model (model 3), the negative correlation between the continuous variable serum apolipoprotein B concentration and lumbar spine BMD was significant in men (*β* = -0.075, 95% CI: −0.103, -0.047, *p* < 0.00001), but not in women (*β* = -0.021, 95% CI: 0.050, 0.007, *p* = 0.14533). Smoothed curve fits of serum apolipoprotein B concentrations stratified by sex with lumbar spine BMD are shown in Figure 3 After covariate transformation for serum apolipoprotein B concentrations (Q1: ≤0.73 g/L,Q2: >0.73, ≤0.89 g/L,Q3: >0.89, ≤1.07 g/L,Q4: >1.07 g/L). In Model 3, with full adjustment for covariates, apolipoprotein B concentrations in the male population were negatively associated with lumbar spine BMD in the Q3 and Q4 intervals, and this association was significant (Q3: β = -0.037, 95% CI: −0.056, -0.017, *p* = 0.00024, Q4: β = -0.056, 95% CI: −0.076, -0.036 (*p* < 0.00001). The trend test found no significant correlation between serum apolipoprotein B and lumbar spine BMD in the Q2 interval. However, overall, there was a trend towards a decrease in lumbar spine BMD with increasing concentrations of apolipoprotein B, and this trend was significant (P trend <0.001). There was also no significant correlation between categorized apolipoprotein B concentrations and lumbar spine BMD in women. The trend was also not statistically significant.

**TABLE 3:** Association between Serum apolipoprotein B (g/L ) and lumbar bone mineral density (subgroup analysis stratified by Sex).

	Model 1	Model 2	Model 3
	β (95% CI) *p* value	β (95% CI) *p* value	β (95% CI) *p* value
Male			
Serum apolipoproteinB ( g/L )	−0.073 (−0.099, −0.047) <0.00001	−0.062 (−0.088, −0.035) <0.00001	−0.075 (−0103, −0.047) <0.00001
Serum apolipoproteinB (g/L ) categories			
Ql (≤ 0.73)	Reference	Reference	Reference
Q2 (> 033, ≤ 0.89)	−0.016 (−0.036,0.004) 0.12013	−0.012 (−0.032, 0.008) 0.23184	−0.018 (−0.038, 0.002) 0.07239
Q3 (> 0.89, ≤ 1.07)	−0.039 (−0.059, −0.020) 0.00009	−0.030 (−0.050, −0.011) 0.00242	−0.037 (−0.056, −0.017) 0.00024
Q4 (> 1.07)	−0.054 (−0.073, −0.035) <0.00001	−0.046 (−0.065, −0.026) <0.00001	−0.056 (−0.076, −0.036) <0.00001
P trend	<0.001	<0.001	< 0.001
Female			
Serum apolipoproteinB ( g/L )	−0.046 (−0.072, −0.019) 0.00072	−0.020 (−0.048,0.008) 0.15724	−0.021(−0.050,0.007) 0.14533
Serum apolipoproteinB (g/L ) categories			
Ql (≤ 0.73)	Reference	Reference	Reference
Q2 (> 033, ≤ 0.89)	−0.027 (−0.045, −0.010) 0.00222	−0.020 (−0.037, −0.002) 0.02579	−0.016 (−0.033,0.001) 0.06211
Q3 (> 0.89, ≤ 1.07)	−0.023 (−0.040, −0.005) 0.01241	−0.011 (−0.028, 0.007) 0.24539	−0.011(−0.029,0.007) 0.24748
Q4 ( >1.07)	−0.033 (−0.051, −0.014) 0.00066	−0.015 (−0.034, 0.005) 0.14195	−0.014 (−0.034,0.006) 0.17086
P trend	0.001	0.244	0.239

Model 1: no covariates were adjusted.

Model 2: Age, Race/ethnicity and Sex were adjusted.

Model 3: Age, Race/ethnicity, Sex, Vigorous recreational activities, Smoked at least 100 cigarettes in life ,Income to poverty ratio, Blood urea nitrogen, Serum calcium, Serum potassium, Serum sodium, Serum phosphorus, Total protein, Serum uric acid, Body mass index.

**FIGURE 4:**
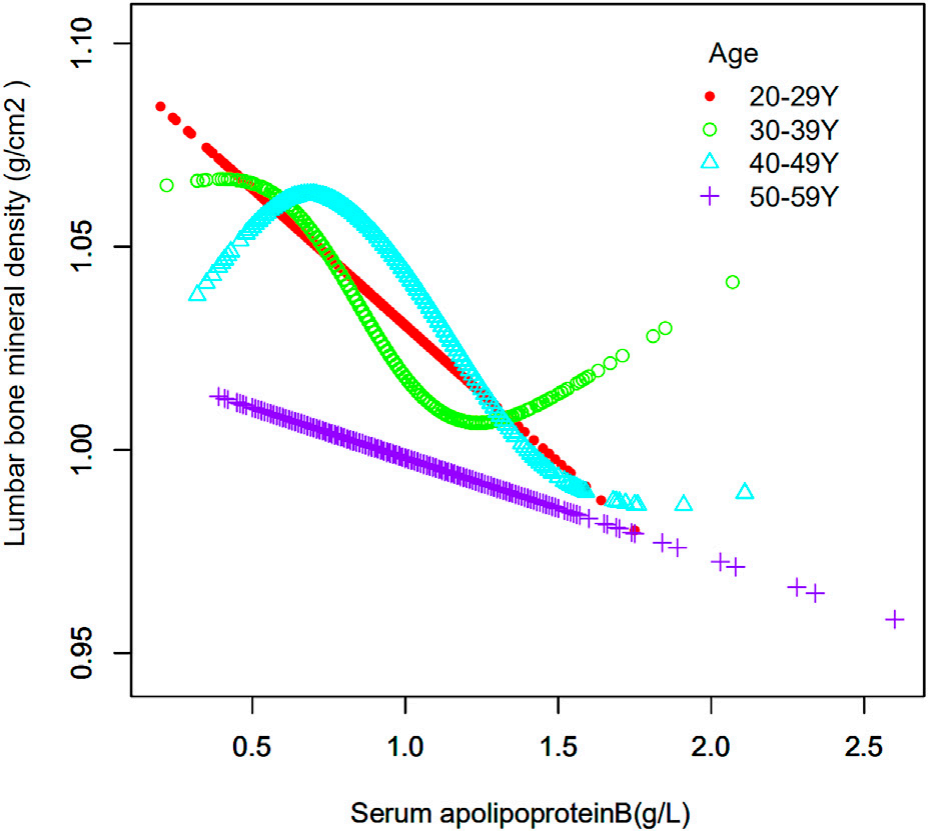
Association between serum apolipoprotein B and lumbar bone mineral density stratified by age. Age, race/ethnicity, sex, vigorous recreational activities, smoked at least 100 cigarettes in life, income to poverty ratio, blood urea nitrogen, serum calcium, serum potassium, serum sodium, serum phosphorus, total protein, serum uric acid, body mass index were adjusted.

The age of the population was analyzed in the strata (Table 4). Their ages were divided into four groups: 20–29, 30–39, 40–49 and 50–59 years. When serum apolipoprotein B concentration was used as a continuous variable, the study found that in the fully adjusted covariate model, 30–39 years (*β* = −0.068, 95% CI: −0.108, −0.027, *p* = 0.00105) and 40–49 years (*β* = −0.065, 95% CI: −0.104, −0.026, *p* = 0.00112) of apolipoprotein B concentrations and lumbar spine BMD, respectively, were negatively correlated with osteoporosis in the cohort, and that this correlation was significant. However, there was no significant correlation between apolipoprotein B concentrations and lumbar spine BMD in individuals aged 20–29 years and 50–59 years. The smoothed curve fits of serum apolipoprotein B concentrations and lumbar spine BMD by age stratification are shown in Figure 4. After categorical transformation of apolipoprotein B concentrations, a negative and significant correlation was found between BMD at age 30–39 years in all apolipoprotein B intervals. The results of the trend test showed a significant decreasing trend in lumbar spine BMD with increasing apolipoprotein B concentrations (P trend <0.001). However, in the 50–59 age group, although there was no significant correlation between apolipoprotein B concentration as a continuous variable and BMD, there was a significant correlation in all concentration ranges of apolipoprotein B. However, the trend towards a decrease in BMD with increasing apolipoprotein B concentration was not significant (P trend = 0.055). In the 20–29- and 40–49-year-old age groups, there was no significant correlation between apolipoprotein B concentrations.

**TABLE 4:** Association between Serum apolipoprotein B (g/L) and lumbar bone mineral density (subgroup analysis stratified by age).

	Model 1	Model 2	Model 3
	β (95% CI) *p* value	β (95% CI) *p* value	β (95% CI) *p* value
20-29Y			
Serum apolipoproteinB (g/L)	−0.043 (−0.084, −0.002) 0.04067	−0.033 (−0.073, 0.008) 0.11121	−0529 (−0.072, 0.014) 0.18136
Serum apolipoproteinB (g/L) categories			
Ql (≤0.73)	Reference	Reference	Reference
Q2 (>0.73, ≤0.89)	−0.012 (−0.033, 0.010) 0.28640	−0.004 (−0.025, 0.017) 0.67903	−0.003 (−0.024, 0.018) 0.79197
Q3 (>0.89, ≤1.07)	−0.024 (−0.047, −0.500) 0.04644	−0.016 (−0.039, 0.007) 0.17227	−0.010 (−0.034, 0.013) 0.39089
Q4 (>1.07)	−0.024 (−0.056, <−0.001) 0.14785	−0.019 (−0.050, 0.013) 0.24178	−0.017 (−0.050, 0.016) 0.32090
P trend	0.032	0.113	0.249
30-39Y			
Serum apolipoproteinB (g/L)	−0.070 (−0.107, −0.032) 0.00031	−0.053 (−0.091, −0.014) 0.00795	−0.068 (−0.108, −0.027) 0.00105
Serum apolipoproteinB (g/L) categories			
Ql (≤0.73)	Reference	Reference	Reference
Q2 (>0.73, ≤0.89)	−0.032 (−0.057, −0.006) 0.01485	−0.028 (−0.053, −0.003) 0.02592	−0.026 (−0.051, −0.001) 0.03840
Q3 (>0.89, ≤1.07)	−0.044 (−0.069, −0.019) 0.00068	−0.036 (−0.061, −0.011) 0.00543	−0.041 (−0.066, −0.016) 0.00160
Q4 (>1.07)	−0.059 (−0.084, −0.033) < 0.00001	−0.049 (−0.075, −0.022) 0.00030	−0.055 (−0.082, −0.028) 0.00007
P trend	< 0.001	< 0.001	<0.001
40-49Y			
Serum apolipoproteinB (g/L)	−0.087 (−0.125, −0.049) < 0.00001	−0.065 (−0.103, −0.028) 0.00072	−0.065 (−0.104, −0.026) 0.00112
Serum apolipoproteinB (g/L) categories			
Ql (≤0.73)	Reference	Reference	Reference
Q2 (>0.73, ≤0.89)	0.002 (−0.029, 0.034) 0.88300	0.011 (−0.020, 0.041) 0.49619	0.012 (−0.019, 0.043) 0.44130
Q3 (>0.89, ≤1.07)	−0.008 (−0.039, 0.023) 0.61452	0.002 (−0.028, 0.033) 0.88555	0.003 (−0.027, 0.034) 0.83398
Q4 (>1.07)	−0.042 (−0.071, −0.012) 0.00629	−0.024 (−0.053, 0.006) 0.11343	−0.021 (−0.052, 0.009) 0.16960
P trend	<0.001	0.022	0.039
50-59Y			
Serum apolipoproteinB (g/L)	−0.025 (−0.064, 0.013) 0.19821	−0.016 (−0.054, 0.022) 0.40255	−0.029 (−0.068, 0.010) 0.14404
Serum apolipoproteinB (g/L) categories			
Ql (≤0.73)	Reference	Reference	Reference
Q2 (>0.73, ≤0.89)	−0.051 (−0.084, −0.019) 0.00205	−0.045 (−0.076, −0.013) 0.00616	−0.048 (−0.080, −0.017) 0.00286
Q3 (>0.89, ≤1.07)	−0.045 (−0.076, −0.015) 0.00385	−0.037 (−0.067, −0.007) 0.01626	−0.048 (−0.078, −0.018) 0.00155
Q4 (>1.07)	−0.041 (−0.071, −0.011) 0.00706	−0.031 (−0.061, −0.002) 0.03651	−0.039 (−0.068, −0.009) 0.00986
P trend	0.059	0.192	0.055

Model 1: no covariates were adjusted

Model 2: Age, Race/ethnicity and Sex were adjusted.

Model 3: Age, Race/ethnicity, Sex, Vigorous recreational activities, Smoked at least 100 cigarettes in life, Income to poverty ratio, Blood urea nitrogen, Serum calciwn, Serum potassium, Serum sodiwn, Serum phosphorus, Total protein, Serum uric acid, Body mass index.

**FIGURE 5:**
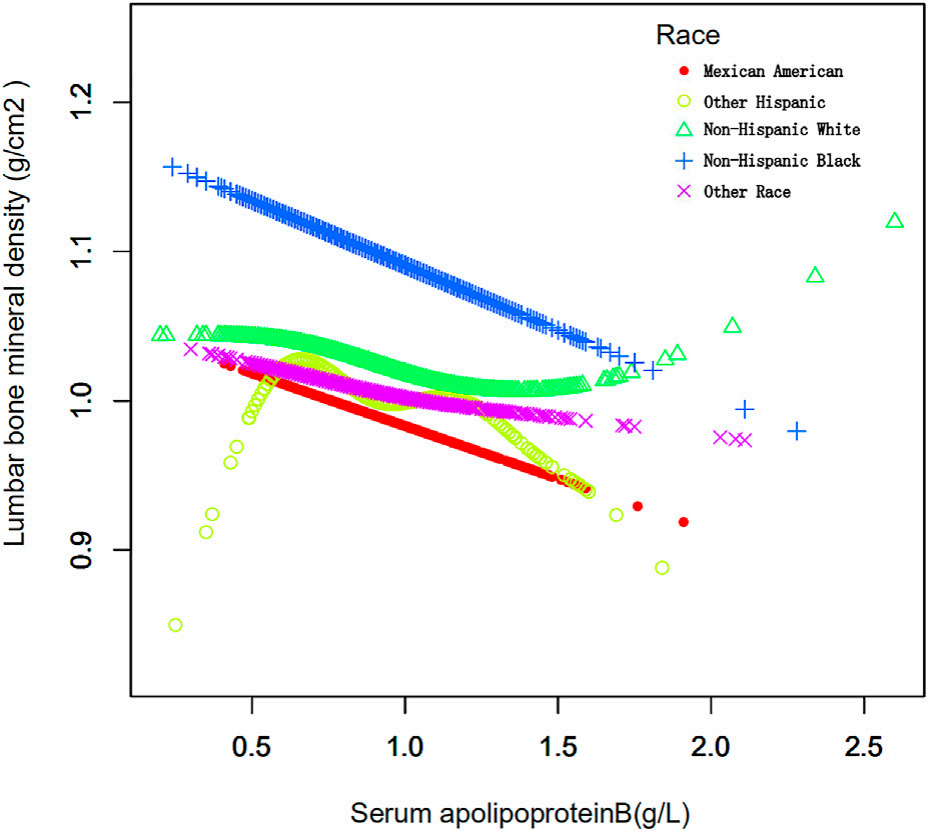
Association between serum apolipoprotein B and lumbar bone mineral density stratified by race. Age, race/ethnicity, sex, vigorous recreational activities, smoked at least 100 cigarettes in life, income to poverty ratio, blood urea nitrogen, serum calcium, serum potassium, serum sodium, serum phosphorus, total protein, serum uric acid, body mass index were adjusted.

The study population was stratified according to race (Table 5). Race was categorized as Mexican American, other Hispanics, non-Hispanic whites, non-Hispanic blacks, or other races. When serum apolipoprotein B concentration was used as a continuous variable, the study found that in the fully adjusted covariate model, Mexican American race (β = −0.069, 95% CI: −0.115, −0.023, *p* = 0.00324), non-Hispanic whites (β = −0.043, 95% CI: −0.076, −0.009), *p* = 0.01205), non-Hispanic blacks (β = −0.089, 95% CI: −0.137, −0.041, *p* = 0.00032), and other races (*β* = −0.065, 95% CI: −0.112, −0.017, *p* = 0.00746), Apolipoprotein B concentration, and lumbar spine BMD were negatively correlated with osteoporosis, and that this correlation was significant. However, the correlation was not significant for the other Hispanics. The smoothed curve fits of serum apolipoprotein B concentrations and age-stratified lumbar spine BMD are shown in Figure 5. Again, after categorical transformation of apolipoprotein B concentrations, in the fully adjusted covariate model, BMD in Mexican American was found to be in the Q4 (*β* = −0.048, 95% CI: −0.080, −0.016, *p* = 0.00376) range for apolipoprotein B. Non-Hispanic White (Q3: β = −0.021, 95% CI: −0.043, 0.001, *p* = 0.06540, Q4: β = −0.033, 95% CI: −0.056, −0.009, *p* = 0.00661) and Non-Hispanic White (Q3: β = −0.021, 95% CI: −0.043, 0.001, *p* = 0.06540, Q4: β = −0.033, 95% CI: −0.056, −0.009, *p* = 0.00661), as well as Non- Hispanic Black (Q3: β = −0.039, 95% CI: −0.073, −0.005, *p* = 0.02399, Q4: β = −0.059, 95% CI: −0.095, −0.024, *p* = 0.00103), in which BMD was significantly and negatively correlated with apolipoprotein B during the Q3 and Q4 intervals. The results of the trend test showed a decrease in lumbar spine BMD with increasing concentrations of apolipoprotein B, with this trend being significant in non-Hispanic whites (P trend = 0.006), non-Hispanic blacks (P trend <0.001), and other races (Q3: β = −0.047, −0.078). There was a significant negative correlation between BMD and apolipoprotein B concentration in the Q3 interval in other Hispanics (95% CI: −0.078, −0.016, *p* = 0.00335).

**TABLE 5:** Association between Serum apolipoproteinB (g/L) and lumbar bone mineral density (subgroup analysis stratified by race).

	Model 1	Model 2	Model 3
	β (95% CI) *p* value	β (95% CI) *p* value	β (95% CI) *p* value
Mexican American			
Serum apolipoproteinB (g/L)	−0.070 (−0.113, −0.028) 0.00109	−0.057 (−0.102, −0.013) 0.01199	−0.069 (−0.115, −0.023) 0.00324
Serum apolipoproteinB (g/L) categories			
Q1 (≤0.73)	Reference	Reference	Reference
Q2 (>0.73, ≤0.89)	−0.022 (−0.053,0.010) 0.18103	−0.016 (−0.048, 0.015) 0.31291	−0.017 (−0.048, 0.014) 0.29319
Q3 (>0.89, ≤1.07)	−0.007 (−0.038, 0.023) 0.63869	0.001 (−0.030, 0.032) 0.93716	−0.007 (−0.038, 0.024) 0.67318
Q4 (>1.07)	−0.052 (−0.082, −0.022) 0.00072	−0.042 (−0.074, −0.010) 0.00950	−0.048 (−0.080, −0.016) 0.00376
P trend	0.002	0.027	0.008
Other Hispanic			
Serum apolipoproteinB (g/L)	−0.040 (−0.095, 0.015) 0.15623	−0.018 (−0.078, 0.041) 0.54694	−0.018 (−0.078, 0.042) 0.55522
Serum apolipoproteinB (g/L) categories			
Q1 (≤0.73)	Reference	Reference	Reference
Q2 (>0.73, ≤0.89)	−0.025 (−0.067, 0.017) 0.23929	−0.019 (−0.061, 0.022) 0.36312	−0.020 (−0.061, 0.021) 0.33571
Q3 (>0.89, ≤1.07)	−0.030 (−0.070, 0.010) 0.14266	−0.022 (−0.062, 0.019) 0.30098	−0.028 (−0.068, 0.012) 0.16840
Q4 (>1.07)	-0.030 (-0.069, 0.009) 0.13199	−0.014 (−0.056, 0.028) 0.50649	−0.019 (−0.061, 0.023) 0.37819
P trend	0.144	0.536	0.354
Non-Hispanic White			
Serum apolipoproteinB (g/L)	−0.043 (−0.073, −0.012) 0.00564	−0.035 (−0.067, −0.003) 0.03168	−0.043 (−0.076, −0.009) 0.01205
Serum apolipoproteinB (g/L) categories			
Q1 (≤0.73)	Reference	Reference	Reference
Q2 (>0.73, ≤0.89)	−0.013 (−0.035, 0.009) 0.23743	−0.012 (−0.033, 0.010) 0.29226	−0.014 (−0.036, 0.007) 0.19858
Q3 (>0.89, ≤1.07)	−0.020 (−0.041, 0.002) 0.07137	−0.017 (−0.038, 0.005) 0.14038	−0.021(−0.043, 0.001) 0.06540
Q4 (>1.07)	−0.033 (−0.054, −0.011) 0.00331	−0.028 (−0.051, −0.005) 0.01642	−0.033 (−0.056, −0.009) 0.00661
P trend	0.003	0.016	0.006
Non-Hispanic Black			
Serum apolipoproteinB (g/L)	−0.103 (−0.148, −0.059) <0.00001	−0.089 (−0.136, −0.043) 0.00016	−0.089 (−0.137, −0.041) 0.00032
Serum apolipoproteinB (g/L) categories			
Q1 (≤0.73)	Reference	Reference	Reference
Q2 (>0.73, ≤0.89)	−0.039 (−0.070, −0.008) 0.01277	−0.033 (−0.064, −0.001) 0.04248	−0.027 (−0.059, 0.004) 0.09228
Q3 (>0.89, ≤1.07)	−0.057 (−0.089, −0.024) 0.00065	−0.045 (−0.078, −0.012) 0.00782	−0.039 (−0.073, −0.005) 0.02399
Q4 (>1.07)	−0.070 (−0.103, −0.038) 0.00003	−0.063 (−0.096, −0.029) 0.00032	−0.059 (−0.095, −0.024) 0.00103
P trend	< 0.001	<0.001	<0.001
Other Race			
Serum apolipoproteinB (g/L)	−0.060 (−0.102, −0.017) 0.00666	−0.055 (−0.100, −0.010) 0.01792	−0.065 (−0.112, −0.017) 0.00746
Serum apolipoproteinB (g/L) categories			
Q1 (≤0.73)	Reference	Reference	Reference
Q2 (>0.73, ≤0.89)	−0.013 (−0.043, 0.016) 0.36321	−0.012 (−0.041, 0.018) 0.43645	−0.016 (−0.045, 0.013) 0.28604
Q3 (>0.89, ≤1.07)	−0.044 (−0.073, −0.014) 0.00393	−0.041(−0.071, −0.010) 0.00984	−0.047 (−0.078, −0.016) 0.00335
Q4 (>1.07)	−0.031(−0.062, 0.001) 0.05399	−0.027 (−0.060, 0.005) 0.10146	−0.033 (−0.067, 0.001) 0.05347
P trend	0.011	0.029	0.014

Model I: no covariates were adjusted.

Model 2: Age, Race/ethnicity and Sex were adjusted.

Model 3: Age, Race/ethnicity, Sex, Vigorous recreational activities, Smoked at least 100 cigarettes in life, Income to poverty ratio, Blood urea nitrogen, Serum calcium, Serum potassium, Serum sodium, Serum phosphorus, Total protein, Serum uric acid, Body mass index.

### 3.3 Association between serum apolipoprotein B and risk of osteopenia or osteoporosis

#### 3.3.1 Overall

In this study, logistic regression models were used to analyze the association between serum apolipoprotein B concentrations and osteopenia or osteoporosis. The results in Table 6 suggest an association between serum apolipoprotein B level as a continuous variable and the risk of osteopenia or osteoporosis. Model 1 of the regression models did not adjust for any covariates; model 2 adjusted for three covariates: sex, age, and race; and model 3 adjusted for all covariates. The results of Model 3 (OR, 1.673; 95% CI, 1.067, 2.624; *p* = 0.025) in the logistic regression model analysis showed that serum apolipoprotein B concentration, a continuous variable, was positively and significantly associated with the risk of osteopenia or osteoporosis. Each unit increase in serum apolipoprotein B level was associated with a 67.3% increased risk of osteopenia or osteoporosis. Classification of serum apolipoprotein B concentrations according to quartiles revealed that serum Apolipoprotein B concentrations were positively associated with the risk of osteopenia or osteoporosis in the Q2 (OR:1.379; 95% CI:1.002, 1.896; *p* = 0.048) and Q4 (OR:1.494; 95% CI:1.071, 2.084; *p* = 0.018) intervals of serum apolipoprotein B. The risks of osteopenia and osteoporosis were positively and significantly correlated. Each unit increase in serum apolipoprotein B concentration in the Q2 interval was associated with a 37.9% increased risk of osteopenia or osteoporosis, and each unit increase in serum apolipoprotein B concentration in the Q4 interval was associated with a 49.4% increased risk of osteopenia or osteoporosis.

**TABLE 6:** Association between Serum apolipoprotein B (g/L ) and the risk of osteopenia or osteoporosis.

	Model 1	Model 2	Model 3
	OR (95% CI) *p* value	OR (95% CI) *p* value	OR (95% CI) *p* value
Serum apolipoprotein B (g/L)	1.981 (1.327, 2.956) 0.001	1.539 (1.003, 2.362) 0.049	1.673 (1.067, 2.624) 0.025
Serum apolipoproteinB (g/L) categories			
Q1 (≤0.73)	Reference	Reference	Reference
Q2 (>0.73, ≤0.89)	1.449 (1.053, 1.996) 0.023	1.350 (0.980, 1.861) 0.066	1.379 (1.002, 1.896) 0.048
Q3 (>0.89, ≤1.07)	1.424 (1.040, 1.949) 0.028	1.241 (0.899, 1.712) 0.189	1.295 (0.938, 1.790) 0.117
Q4 (>1.07)	1.701 (1.244, 2.326) 0.001	1.418 (1.027, 1.957) 0.034	1.494 (1.071, 2.084) 0.018

Model 1: no covariates were adjusted

Model 2: Age, Race/ethnicity and Sec were adjusted

Model 3: Age, Race/ethnicity, Sex, Vigorous recreational activities, Smoked at least 100 cigarettes in life, Income to poverty ratio, Blood urea nitrogen, Serum calcium, Serum potassium, Serum sodium, Serum phosphorus, Total protein, Serum uric acid, Body mass index.

#### 3.3.2 Stratification by sex, age and race

The study stratified the population according to sex (Table 7). Serum apolipoprotein B concentration was used as a continuous variable for risk factor analysis. In a fully adjusted covariate model (model 3), the positive association between the continuous variable serum apolipoprotein B concentration and the risk of osteopenia or osteoporosis was significant in men (OR: 2.254; 95% CI: 1.208, 4.206; *p* = 0.011), and each unit increase in serum apolipoprotein B concentration was associated with a 125.4% increase in the risk of osteopenia or osteoporosis. When apolipoprotein B concentration quartiles were classified, there was a significant positive correlation only in the Q4 interval (OR: 2.011; 95% CI: 1.238, 3.267; *p* = 0.005), and each unit increase in serum apolipoprotein B concentration was associated with a 101% increased risk of osteopenia or osteoporosis. In the female population, apolipoprotein B was not significantly associated with the risk of osteopenia or osteoporosis, either as a continuous or categorical variable.

**TABLE 7:** Association between Serum apolipoprotein B (g/L) and and the risk of osteopenia or osteoporosis. (subgroup analysis stratified by sex).

	Model 1	Model 2	Model 3
	OR (95% CI) *p* value	OR (95% CI) *p* value	OR (95% CI) *p* value
Male			
Serum apolipoproteinB (g/L)	2.243 (1.267, 3.972) 0.006	1.816 (1.008, 3.269) 0.047	2.254 (1.208, 4.206) 0.011
Serum apolipoproteinB (g/L) categories			
Q1 (≤0.73)	Reference	Reference	Reference
Q2 (>0.73, ≤0.89)	1.504 (0.903, 2.505) 0.117	1.396 (0.838, 2.325) 0.201	1.529 (0.917, 2.548) 0.103
Q3 (>0.89, ≤1.07)	1.539 (0.942, 2.514) 0.085	1.361 (0.826, 2.242) 0.226	1.503 (0.908, 2.489) 0.113
Q4 (>1.07)	1.995 (1.251, 3.181) 0.004	1.692 (1.058, 2.706) 0.028	2.011 (1.238, 3.267) 0.005
Female			
Serum apolipoproteinB (g/L)	1.918 (1.080, 3.408) 0.026	1.036 (0.546, 1.965) 0.914	1.120 (0.564, 2.223) 0.746
Serum apolipoproteinB (g/L) categories			
Q1 (≤0.73)	Reference	Reference	Reference
Q2 (>0.73, ≤0.89)	1.439 (0.954, 2.171) 0.083	1.261 (0.826, 1.924) 0.283	1.227 (0.807, 1.866) 0.339
Q3 (>0.89, ≤1.07)	1.389 (0.921, 2.097) 0.117	1.058 (0.683, 1.638) 0.801	1.063 (0.690, 1.637) 0.782
Q4 (>1.07)	1.541 (0.989, 2.402) 0.056	1.013 (0.632, 1.625) 0.957	1.055 (0.652, 1.706) 0.828

Model 1: no covariates were adjusted.

Model 2: Age, Race/ethnicity and Sex were adjusted.

Model 3: Age, Race/ethnicity, Sex, Vigorous recreational activities, Smoked at least 100 cigarettes in life, Income to poverty ratio, Blood urea nitrogen, Serum calcium, Serum potassium, Serum sodium, Serum phosphorus, Total protein, Serum uric acid, Body mass index.

Age-stratified analysis of the study population was performed (Table 8). Serum apolipoprotein B concentration was used as a continuous variable. In the model fully adjusted for covariates (model 3), only the positive association between serum apolipoprotein B concentration and risk of osteopenia or osteoporosis was significant in the 40–49 age group (OR:3.204; 95% CI:1.32, 7.779; *p* = 0.01), and each unit increase in serum apolipoprotein B concentration increased the risk of osteopenia or osteoporosis by 220% for each unit increase in serum apolipoprotein B concentration. When quartiles of apolipoprotein B concentrations were classified, each unit increase in serum apolipoprotein B concentration in the Q3 and Q4 intervals increased the risk of osteopenia or osteoporosis by 92% and 93%, respectively, in the 30–39 age group. The risk of osteopenia or osteoporosis increased by 113% in the Q2 interval in the 50–59 to age group.

**TABLE 8:** Association between Serum apolipoprotein B (g/L) and and the risk of osteopenia or osteoporosis (subgroup analysis stratified by age).

	Model 1	Model 2	Model 3
	OR (95% CI) *p* value	OR (95% CI) *p* value	OR (95% CI) *p* value
20-29Y			
Serum apolipoproteinB (g/L)	0.699 (0.267, 1.829) 0.466	0.633 (0.236, 1.701) 0.365	0.530 (0.186,1.51) 0.234
Serum apolipoproteinB (g/L) categories			
Ql(≤0.73)	Reference	Reference	Reference
Q2(>0. 73, ≤0.89)	0.777 (0.454, 1.332) 0.360	0.725 (0.419, 1.252) 0.248	0.698 (0.405, 1.204) 0.196
Q3(>0.89, ≤1.07)	1.062 (0.595, 1.897) 0.839	1.012 (0.557, 1 838) 0.969	0.877 (0.486,1.583) 0.664
Q4(>1.07)	0.601 (0.299, 1.210)0.154	0.564(0.282, 1.127) 0.105	0.517 (0.244, 1.097)0.086
30-39Y			
Serum apolipoproteinB (g/L)	2.174 (0.979, 4.827) 0.056	1.825(0.779,4.279)0.166	2.158 (0.893,5.213)0.087
Serum apolipoproteinB (g/L) categories			
Ql(≤0.73)	Reference	Reference	Reference
Q2(>0. 73, ≤0.89)	1.922 (0.996, 3.711) 0.051	1.851(0.952, 3.596)0.069	1.799 (0.938, 3.450) 0.077
Q3(>0. 89, ≤1.07)	2.012 (1.081, 3.745) 0.027	1.845(0.982,3.468)0.057	1.929 (1.038, 3.583)0.038
Q4(>1.07)	2.046 (1.089, 3.847) 0.026	1.816 (0.956, 3.451) 0.068	1.933 (1.020,3.663)0.043
40-49Y			
Serum apolipoproteinB (g/L)	3.354 (1.459, 7.711) 0.004	2.989 (1.281, 6.974) 0.011	3.204 (1.320, 7.779) 0.010
Serum apolipoproteinB (g/L) categories			
Ql(≤0.73)			
Q2(>0. 73, ≤0.89)	1.423 (0.685, 2.956) 0.344	1.337 (0.638, 2.802) 0.441	1.410 (0.666,2.981) 0.369
Q3(>0. 89, ≤1.07)	1.150 (0.565,2.339) 0.700	1.067 (0.522, 2.182)0.858	1.164 (0.554, 2.448) 0.688
Q4(>1.07)	2.063 (1.058,4.022)0.034	1.831 (0.932, 3.599) 0.079	1.964 (0.961, 4.013) 0.064
50-59Y			
Serum apolipoproteinB (g/L)	0.976 (0.456, 2.092)0.951	0.867(0.389,1.929)0.726	1.248 (0.557, 2.797) 0.590
Serum apolipoproteinB (g/L) categories			
Q1(≤0.73)			
Q2(>0. 73, ≤0.89)	2.215 (1.023,4.798)0.044	2.043(0.914,4.567)0.082	2.382 (1.074,5.286) 0.033
Q3(>0.89, ≤1.07)	1.551 (0.726,3.313) 0.257	1.395(0.632,3.078)0.410	1.805 (0.823, 3.957)0.140
Q4(>1.07)	1.507 (0.712, 3.191) 0.283	1.324(0.611,2.872) 0.477	1.743 (0.803, 3.786) 0.160

Model 1: no covariates were adjusted.

Model 2: Age, Race/ethnicity and Sex were adjusted.

Model 3: Age, Race/ethnicity, Sex, Vigorous recreational activities, Smoked at least 100 cigarettes in life, Income to poverty ratio, Blood urea nitrogen, Serum calcium, Serum potassium, Serum sodium, Serum phosphorus, Total protein, Serum uric acid, Body mass index.

Stratified analysis of the population by ethnicity was performed (Table 9). Serum apolipoprotein B concentration was used as a continuous variable. In the model with full adjustment for covariates (model 3), only the positive association between serum apolipoprotein B concentration and risk of osteopenia or osteoporosis was significant in the Mexican American and Non-Hispanic Black populations, with each unit increase in serum apolipoprotein B concentration increasing the risk of osteopenia or osteoporosis by 547% and 469%. The risk of osteoporosis was increased by 547% and 469% for each unit increase in serum apolipoprotein B concentration. When quartiles of apolipoprotein B concentrations were classified, the Mexican American group showed significant results only in the Q4 interval, with each unit increase in serum apolipoprotein B concentrations associated with a 199% increase in the risk of osteopenia or osteoporosis. The non-Hispanic black group showed significant correlations in the Q1, Q2, and Q3 intervals, with each unit increase in serum apolipoprotein B concentration increasing the risk of osteopenia and osteoporosis by 160%, 133%, and 390%, respectively.

**TABLE 9:** Association between Serum apolipoprotein B (g/L) and the risk of osteopenia or osteoporosis (subgroup analysis stratified by race).

	Model 1	Model 2	Model 3
	OR (95% CI) *p* value	OR (95% CI) *p* value	OR (95% CI) *p* value
Mexican American			
Serum apolipoproteinB (g/L)	3.441 (1.566,7.557)0.002	4.275 (1.827, 10.002) 0.001	6.479 (2.507, 16.747) < 0.001
Serum apolipoproteinB (g/L) categories			
Q1 (≤ 0.73)	Reference	Reference	Reference
Q2 (> 0.73, ≤ 0.89)	1.147 (0.584,2.253) 0.691	1.116 (0.556, 2.239) 0.758	1.145 (0.562, 2.335) 0.709
Q3 (> 0.89, ≤ 1.07)	1.438 (0.772, 2.679) 0.252	1.418 (0.758, 2.653) 0.274	1.616 (0.841,3.106) 0.150
Q4 (> 1.07)	2.139 (1.176, 3.291) 0.013	2.383 (1.272, 4.466) 0.007	2.995 (1.519, 5.904) 0.002
Other Hispanic			
Serum apolipoproteinB (g/L)	3.301 (1.063, 10.248) 0.039	2.293 (0.635, 8.276) 0.205	2.066 (0.488, 8.753) 0.325
Serum apolipoproteinB (g/L) categories			
Q1 (≤ 0.73)	Reference	Reference	Reference
Q2 (> 0.73, ≤ 0.89)	1.173 (0.547, 2.517) 0.682	0.993 (0.460, 2.146) 0.986	0.934 (0.423, 2.062) 0.865
Q3 (> 0.89, ≤ 1.07)	0.894 (0.416, 1.922) 0.775	0.750 (0.333, 1.686) 0.486	0.754 (0.330, 1.725) 0.504
Q4 (> 1.07)	2.095 (1.051, 4.173) 0.036	1.576 (0.748, 3.324) 0.232	1.527 (0.665, 3.503) 0.318
Non-Hispanic White			
Serum apolipoproteinB (g/L)	1.354 (0.733, 2.499) 0.333	1.063 (0.56, 2.019) 0.852	1.155 (0.589, 2.267) 0.674
Serum apolipoproteinB (g/L) categories			
Q1 (≤ 0.73)	Reference	Reference	Reference
Q2 (> 0.73, ≤ 0.89)	1.411 (0.888, 2.242) 0.146	1.338 (0.845, 2.121) 0.215	1.380 (0.870, 2.187) 0.171
Q3 (> 0.89, ≤1.07)	1.266 (0.796, 2.013) 0.319	1.144 (0.715, 1.833) 0.574	1.193 (0.741, 1.921) 0.467
Q4 (> 1.07)	1.306 (0.808, 2.111) 0.276	1.107 (0.681, 1.797) 0.682	1.173 (0.708, 1.943) 0.536
Non-Hispanic Black			
Serum apolipoproteinB (g/L)	6.278 (3.043, 12.953) < 0.001	5.263 (2.498, 11.087) < 0.001	5.697 (2.499, 12.986) < 0.001
Serum apolipoproteinB (g/L) categories			
Q1 (≤ 0.73)	Reference	Reference	Reference
Q2 (> 0.73, ≤ 0.89)	3.230 (1.514, 6.890) 0.002	2.800 (1.299, 6.032) 0.009	2.603 (1.217, 5.566) 0.014
Q3 (> 0.89, ≤ 1.07)	3.075 (1.421, 6.656) 0.004	2.441 (1.106, 5.384) 0.027	2.332 (1.052, 5.171) 0.037
Q4 (> 1.07)	5.725 (2.773,11.818) < 0.001	4.833 (2.31, 10.114) < 0.001	4.904 (2.296, 10.474) < 0.001
Other Race			
Serum apolipoproteinB (g/L)	1.473 (0.629, 3.450) 0.373	1.424 (0.529, 3.839) 0.484	1.7 (0.533, 5.418) 0.370
Serum apolipoproteinB (g/L) categories			
Q1 (≤ 0.73)	Reference	Reference	Reference
Q2 (> 0.73, ≤ 0.89)	1.230 (0.667, 2.268) 0.508	1.143 (0.622, 2.101) 0.667	1.226 (0.659, 2.282) 0.520
Q3 (> 0.89, ≤ 1.07)	1.699 (0.960, 3.005) 0.069	1.639 (0.897, 2.993) 0.108	1.830 (1.000, 3.349) 0.050
Q4 (> 1.07)	1.246 (0.680, 2.281) 0.476	1.197 (0.608, 2.354) 0.603	1.311 (0.596, 2.882) 0.501

Model 1: no covariates were adjusted.

Model 2: Age, Race/ethnicity and Sex were adjusted.

Model 3: Age, Race/ethnicity, Sex, Vigorous recreational activities, Smoked at least 100 cigarettes in life, Income to poverty ratio, Blood urea nitrogen, Serum calcium, Serum potassium, Serum sodium, Serum phosphorus, Total protein, Serum uric acid, Body mass index.

## 4 Discussion

The primary objective of this study was to examine the association between apolipoprotein B and lumbar spine BMD and the risk of osteopenia or osteoporosis. In this cross-sectional study, a nationally representative US population aged 20–59 years was used to examine the association between serum apolipoprotein B and lumbar spine BMD and osteopenia or osteoporosis. Data were selected from the central data of the US NHANES database. Our study showed a significant negative association between serum apolipoprotein B concentration and lumbar spine BMD in the total population, and a significant positive association with the risk of osteopenia or osteoporosis. After stratifying the group by sex, this correlation was significant in males but not in females. After stratification by age, serum apolipoprotein B concentrations, and the risk of lumbar spine BMD, osteopenia, and osteoporosis was correlated to varying degrees in different age groups. The negative correlation between serum apolipoprotein B concentration and lumbar spine BMD and the positive correlation between the risk of osteopenia or osteoporosis were more pronounced in the 30–39 years and 50–59 years age groups. After stratifying the groups by ethnicity, there was a significant negative association between serum apolipoprotein B concentrations and lumbar spine BMD, and a significant positive association between the risk of osteopenia or osteoporosis in the Mexican American and non-Hispanic black groups. Therefore, these findings suggest that these associations are influenced by sex, age, and ethnicity. The present study is the first to examine the association between serum apolipoprotein B concentrations and BMD and the risk of osteopenia or osteoporosis.

In a study by Kim et al. (2010), there was a significant negative correlation between fat mass and BMD, particularly in men. However, the findings of (Qiao et al., 2020) again suggest that adult obese patients have a higher bone density in the lumbar spine and femoral neck than individuals with healthy weights, which again contradicts the results of our experiment. It is possible that differences in the population inclusion and exclusion criteria contributed to the similarities and differences in the results of the two studies. The protein fraction of lipoproteins is called apolipoprotein (Apo) (Mooser et al., 1997), and has important physiological functions in lipoprotein metabolism (Florea et al., 2022). Apolipoprotein B is synthesized in the liver (Bell et al., 2011) and is the main structural protein of LDL (Yaseen et al., 2021), accounting for most of the total protein content of LDL, with its measurement directly reflecting LDL levels (Sun et al., 2020). Elevated obesity (weight overload) leads to elevated levels of apolipoprotein B in the blood (Manapurath et al., 2022), which decrease with exercise, vegetarianism, and hypolipidemia (Hostmark et al., 1993; Gao et al., 2018; Stanton et al., 2022). In hyperlipidemia, diabetes, and atherosclerosis, this is often accompanied by an increase in apolipoprotein B (Glowinska et al., 2003), with changes in apolipoprotein B concentrations in the blood therefore possibly being indicative of health problems caused by lipid metabolism. It has been suggested that obesity leads to a reduction in bone mineral density (Bagi et al., 2018), and that a high-fat environment leads to disruption of lipid metabolism and impaired differentiation of bone marrow mesenchymal stem cells (Bi et al., 2021); however, biochemical tests for hyperlipidemia are often limited to plasma triglycerides and cholesterol (Adeyanju et al., 2022). Apolipoprotein B has rarely been used as an independent indicator of hyperlipidemia or as a predictor of changes in bone mineral density. Therefore, in the present study, we attempted to find a new indicator of apolipoprotein B as a predictor of changes in bone mineral density and the risk of reduced bone mass or osteoporosis. There is very limited evidence in previous studies directly linking apolipoprotein B and lumbar spine BMD (Yerges-Armstrong et al., 2013). Therefore, there was a strong rationale to conduct this study. It would be in the interest of the vast majority of patients if serum apolipoprotein B concentrations were routinely used as an indicator in clinical investigations to assess the risk of osteopenia or osteoporosis based on the results of these investigations. It is possible to reduce the risk of osteopenia or osteoporosis by adjusting one’s diet and lifestyle according to serum apolipoprotein B concentrations at the time of the test. This is of clinical importance to patients.

This study has several strengths. First, the database we used is nationally representative, and the NHANES database uses a standardized and uniform protocol for data collection. Therefore, the reliability of the included data is high. The weighted data are visually representative of the national population data. Second, this study assessed the association between serum apolipoprotein B concentrations and lumbar spine BMD using three models with multiple regression equations and the association between serum apolipoprotein B concentrations and the risk of osteopenia or osteoporosis using logistic regression models. Stratified population analyses were used to assess differences in serum apolipoprotein B concentrations and risk of lumbar spine BMD and osteopenia or osteoporosis by sex, age group, and ethnicity. Although the database used in this study is large and comprehensive, it has some limitations. First, this was a cross-sectional study; therefore, the causal relationship between the independent and dependent variables was uncertain. Second, the NHANES database was collected only once, which does not exclude the bias that arose at the time of data collection.

## Data Availability

The datasets for this study can be found at www.cdc. gov/nchs/nhanes/.
